# Treatment of distal femur aseptic nonunion after lateral locking plate fixation: Results of medial custom made plating and free fibula flap transfer using CAD-CAM technology

**DOI:** 10.1016/j.jham.2024.100169

**Published:** 2024-10-12

**Authors:** Vittorio Ramella, Gianluca Canton, Micol Dussi, Cristina Formentin, Veronica Scamacca, Filippo Bagnacani, Trobec Belinda, Luca Spazzapan, Luigi Troisi, Laura Grezar, Giovanni Papa, Luigi Murena

**Affiliations:** a Plastic, Reconstructive and Aesthetic Surgery Unit, Cattinara Hospital - ASUGI, Department of Medical, Surgical and Life Sciences, Trieste University, Trieste, Italy; b Orthopaedics and Traumatology Unit, Cattinara Hospital - ASUGI, Department of Medical, Surgical and Life Sciences, Trieste University, Trieste, Italy; c Head of the Reconstructive Microsurgery Service, University Department of Hand Surgery & Rehabilitation - San Giuseppe Hospital - IRCCS MultiMedica Group, Milan, Italy

**Keywords:** CAD-CAM, Custom made, Nonunion, Distal femur, Free fibula flap

## Abstract

**Background:**

Aim of the present paper is to report the preliminary results of CAD-CAM (Computer-Aided Design - Computer-Aided Manufacturing) technology application to distal femur nonunion treatment with free fibula flap, custom made medial plating and maintenance of a stable lateral locking plate.

**Methods:**

Two cases of distal femur nonunion that occurred after lateral locking plating were treated and prospectively followed-up. Surgical planning followed the same preoperative protocol adopted for mandibular CAD-CAM reconstruction. Wide cutting sections were planned to obtain radical debridement. The tailored custom-made plate, a 3D rendering of bone defect and the cutting guides were produced and sterilized. Surgical intervention was conducted by steps (medial approach, bone resection, recipient vessels isolation, fibula harvesting and cutting, plate-fibula construct assembly, microvascular anastomosis, final fixation).

**Results:**

The mean follow-up was 13 (12–15) months. Bone union was achieved in both cases at mean 3.1 months. Full weight bearing without referred pain or discomfort was reached in both cases at mean 8,5 months (range 7–10). No complications occurred.

**Conclusions:**

CAD-CAM technology proved to be useful and reliable in custom made medial plating combined with free fibula transfer for the treatment of distal femur nonunion after lateral locking plating.

**Trial registration:**

none.

## 1 Introduction

Distal femur fractures account for 1 % of all fractures with a rate of non-unions that range from 5 to 10 % of cases. There are several recognized risk factors for nonunion, usually divided in biological and mechanical factors. Biological factors include open fracture, comminution and fracture gap, fracture location (especially regarding local vascularity), open reduction, infection, drug intake (NSAIDs, steroids, anticoagulants, chemotherapeutic drugs), alcohol abuse, diabetes mellitus, smoking, genetic disorders, and chronic inflammatory diseases. Mechanical factors may be related to both unstable fixation and inadequate stiffness and/or reduction with respect to fracture type.^1^^,^^2^ Surgical treatment for nonunion generally requires hardware removal, debridement of fibrous tissue and non-viable bone, bone transplant and adequate fixation.^3^^,^^4^ In some cases of distal femur fracture fixation with lateral locking plates, metaphyseal nonunion occurs without hardware failure. These cases are characterized by a variable amount of metaphyseal bone resorption, a maintained overall alignment and an adequate mechanical stability of the plate. Risk factors for this specific form of nonunion, besides other commonly recognized factors, are primarily metaphyseal comminution^5^ and excessive stiffness of fracture fixation.^6^^,^^7^ In these cases, the surgeon might choose not to replace the stable lateral plate, treating bone resorption with bone grafting and adding mechanical stability on the medial side with various described techniques.^8^^,^^9^ However, a quite relevant failure rate for these treatment strategies is reported, with bone graft resorption being the major concern.^10^, ^11^, ^12^ The use of a vascularized flap could be the solution to avoid bone graft failure, due to the well-known advantages of this technique. In detail, a free fibula flap in this scenario would carry at once vascularity, osteogenetic progenitors and a combined osteoinductive and osteoconductive stimulus, together with an aid in overall mechanical stability.^13^ Nonetheless, the procedure is technically demanding especially if concomitant medial plating is required to add stability.

A possible solution to overcome the technical difficulties of combining free-fibula flap transfers with plating is offered by the application of CAD-CAM (Computer-Aided Design - Computer-Aided Manufacturing) technology. Literature reports good results in some non-orthopedic applications such as mandibular reconstruction with free fibula flap in patients with squamous cell carcinoma and mandibular osteoradionecrosis.^14^ Aim of the present paper is to report the preliminary results of CAD-CAM technology application to distal femur nonunion treatment with free fibula flap, custom made medial plating and maintenance of a stable lateral locking plate.

## 2 Material and methods

**Fig. 1:**
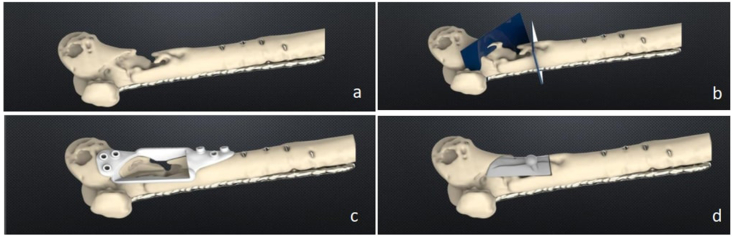
Pre-operative planning, part 1. 3d rendering of bone defect and planned resection of non viable bone (a,b). CAD-CAM cutting guide (c) and defect template (d) planned to allow for precise free-fibula bone grafting.

Two cases of distal femur nonunion that occurred after lateral locking plating were treated and prospectively followed-up between December 2016 and January 2018. Both cases were characterized by metaphyseal bone resorption, maintained alignment and adequate mechanical stability of the lateral plate. All patients signed the informed consent before being included in the study. The study was performed in accordance with the Ethical standards of the 1975 Declaration of Helsinki, as revised in 2000. None of the two patients presented with osteomyelitis, infected nonunion, previous traumatic damage to the leg or the *peronea magna* variant of leg vascularization,^15^ identified as exclusion criteria. Patients’ demographics, mechanism of injury and previous surgery were collected. Surgical planning followed the same preoperative protocol adopted for mandibular CAD-CAM reconstruction.^14^ Angiography-CT scans of the lower limbs were obtained in each patient in order to classify the type of defect and to assess the lower leg vascularization. An iCT 256 Philips CT scanner (Eindoven, NL) has been used. The DICOM files (256x0.625 - 64-slice, high resolution) were sent to the biomedical engineering company (Sintac srl, Rovereto, Italy) to perform the three-dimensional rendering (Fig. 1a). The orthopedic surgeons, plastic reconstructive surgeons and the engineers involved in the surgical planning attended a videoconference to evaluate the amount of bone to be removed at the nonunion site and the length of fibula to be harvested. A radical debridement of necrotic bone was planned in all patients, thus wide cutting sections were designed to achieve this goal (Fig. 1b and c,d).

**Fig. 2:**
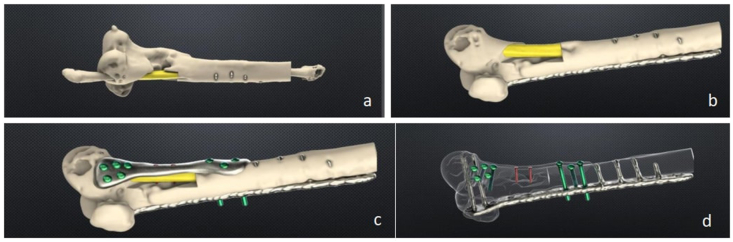
Pre-operative planning, part 2. Planning of measured fibula resection to fit the resected area (a,b). Shaping of the plate to fit the anteromedial distal femur anatomy (c). Screws direction (d) with planned interaction with previous plates and screws.

The tailored custom-made plate with dedicated screws for femur and fibula fixation, a 3D rendering of bone defect and the cutting guides for both recipient and donor sites were then produced and sterilized (Fig. 2).

**Fig. 3:**
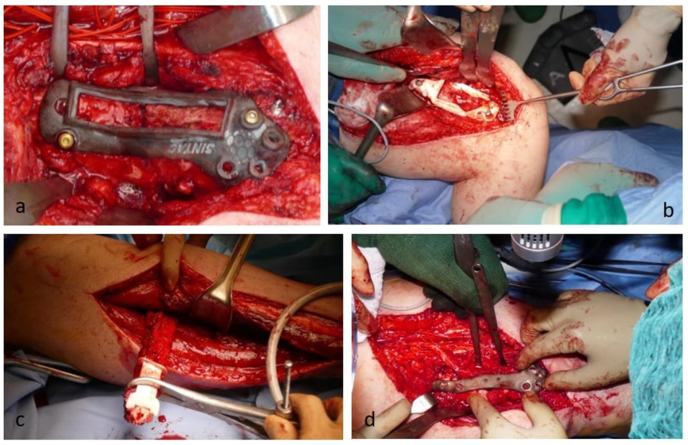
The CAD/CAM cutting guide is positioned on the antero-medial distal femur according to pre-operative planning to allow wide controlled resection of the non-union site (a,b). Fibula flap harvest with the custom-made resection guide (c). Final aspect of medial custom-made plating, free fibula transfer and cancellous bone allograft added with BMAC (d).

**Fig. 4:**
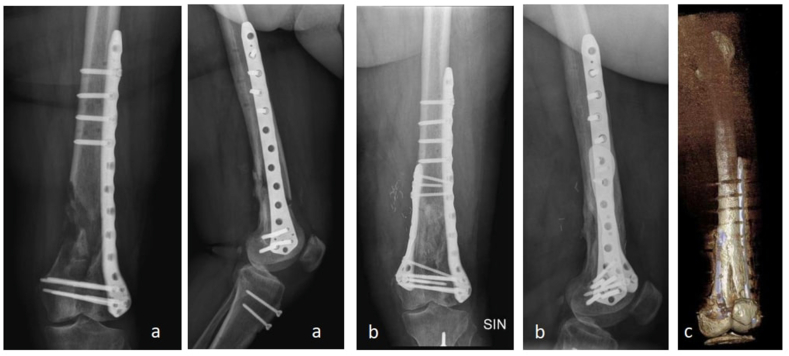
Clinical case n°1. Pre-op X-rays (a), post op X-rays (b) and 3D CT scan (c) results at 12 months.

Surgical intervention was conducted by steps. First, the non-union site was reached through a medial approach and debrided using the previously determined CAD/CAM cutting guides in order to remove the exact amount of necrotic bone and fibrous tissue (Fig. 3a and b) needed to seat the implant and the graft. The previously implanted lateral locking plate was not removed. The descending genicular vessels were identified and selected as recipient vessels. Then, the free fibula flap was harvested and cut using the dedicated cutting guides (Fig. 3c). The resulting graft fitted perfectly into the femoral bone defect resulting after resection of the non-union area, thus creating precise contact and compression with the remaining femoral stumps. The ad hoc prefabricated plate was fixed to the harvested fibula before cutting the vascular pedicle to minimize the ischemia time. Subsequently, microvascular anastomosis was performed (mean ischemia time 30 min). Finally, the flap with the mounted prefabricated tailored plate was fixed to the femur by screws inserted through the previously planned holes. Once the medial column was reconstructed with the described technique, cancellous allograft enriched with bone marrow aspirate concentrate (BMAC, Joint srl, Mestre, Italy) and fibrin glue was placed to fulfill the residual metaphyseal bone loss (Fig. 3d). Surgical wound was closed in layers and a subcutaneous drainage was placed. After surgery, active and passive mobilization were allowed and the patient was instructed to ambulate with 50% weight bearing for 3 weeks. Post-operative X-rays were taken after one, three, six and twelve months. A 3D-CT scan was also performed at six and twelve postoperative months (Fig. 4). Time to union, time to full weight bearing, resumption of normal life activities and any complication including 175 donor site morbidity were recorded.

## 3 Results

The overall data about the patients are collected in Table 1. The mean follow-up was 13 months (range 12–15). Bone union was achieved in both cases at mean 3.1 months (range 3–3.2). Full weight bearing without referred pain or discomfort was reached in both cases at mean 8,5 months (range 7–10).

**Table 1:** Clinical characteristics and outcomes for each patient.

	Patient 1	Patient 2
**Sex**	F	F
**Age (yo)**	58	74
**Side**	Left	Right
**Mechanism of injury**	Fall from 7 m	Street injury (pedestrian)
**Comorbidity**	None	Diabetes, mild cardiovascular disease
**Previous surgery**	DCO(spanning EXFIX), ORIF	ORIF
**FVFG length (cm)**	6.2	6.1
**Time to radiological confirmation of bone union (months)**	3.2	3
**Time to partial weight bear (days)**	21	23
**Time to full weight bear (months)**	7	10
**Follow Up (months)**	15	12
**Recipient site complications**	None	None
**Donor site complications**	None	None
**Pain**	Resolved	Resolved

**Fig. 5:**
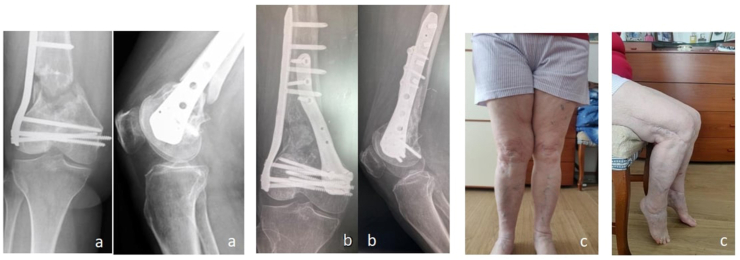
Clinical case n°2. Pre-op X-rays (a), Post-op x-rays (b) and clinical (c) results at 15 months.

The X-rays and 3D CT scan performed one year after surgery showed high quality bone formation (Fig. 4, Fig. 5). No patient reported donor site morbidity. All patients were able to resume normal life activities without complaints.

The technology offers an improved surgical precision defining the exact mechanical position and dimension of the custom-made medial additional plate and of the FFVG and its vascular pedicle. However, further studies are needed to confirm the validity and reproducibility of the described treatment and to demonstrate the cost-savings offered by the application of CAD/CAM technology in the treatment of these particular non-unions.

In our hands it allowed a safe, reliable and timesaving treatment, with a 100 % resolution of the preoperative clinical condition and a return to everyday life with full weight bearing in both cases. The main limit of our study is the low number of patients involved, which is related to the rarity of the condition treated.

## 4 Discussion

Long bone non-unions are difficult to manage, with long-lasting treatment and expensive implications. Inappropriate mechanical environment, fracture site biology impairment and patient host factors represent recognized risk factors for development of bone non-union.^2^ Distal femur fractures account for 4–6% of all femoral fractures, with a rate of non-union from 5 to 10 %.^16^^,^^17^ Treatment of distal femur fracture with Lateral Locking Plate (LLP) has become widely popular. In literature non-union rates following this treatment strategy range from 6 % to 21 %.^17^ In a systematic review of Wang et al., the authors identify some risk factors which contribute to developing distal femur non-union after fixation with LLP: stainless steel plate material, high construct rigidity scores and purely locking screw constructs.^18^ Conventionally, atrophic non-union treatment consists of debridement of fibrous tissue, hardware removal, new osteosynthesis and biological stimulation by bone grafting and/or growth factor application.^19^^,^^20^ In presence of poor alignment because of previous fixation failure, hardware removal and fixation revision are paramount.^21^ However, in some cases of distal femur extrarticular fracture fixation with LLP, non-union occurs without hardware failure. These cases are characterized by a variable amount of metaphyseal bone resorption with residual gap, a maintained overall alignment and an adequate mechanical stability of the plate.^22^ In these cases, the surgeon might choose not to replace the stable lateral plate and to treat instead the bone resorption with bone graft and by adding mechanical stability on the medial side. In a study by Holzman et al., medial plate positioning and bone grafting in distal femur fracture non-union (with an intact and stable LLP), has proved to be a good method to achieve bone union.^8^ However, some authors suggested both isolated bone grafting and medial plating alone might be successful.^22^ According to other authors, the lateral locking plate should always be revised.^8^ Thus, while achieving structural stability and enhancing biology with the same procedure is recognized to be the mainstay of treatment, the ideal strategy is still to be defined. Free vascularized fibula graft (FVFG) provides a high quality and a highly vascularized bone segment^23^^,^^24^ which overcomes the compromised vascularity of the recipient site. The high potential of osteoconduction and osteoinduction is combined with a relatively low rate of donor-site morbidity. Moreover, the wide segment of healthy cortical bone with its wide contact surface contributes to mechanical stability. Nonetheless, FVFG presents some disadvantages, mainly fibula stress fracture, temporary leg muscle weakness^23^ and possible ankle instability if at least 6 cm of distal fibula are not preserved.^24^ Moreover, the procedure is technically demanding, requires microsurgical anastomosis and in distal femur non-union cases it becomes especially cumbersome when an additional medial plate is required to add mechanical stability. In the authors opinion, the application of CAD-CAM technology to distal femur non-unions is a feasible solution to overcome these technical difficulties. This technique was developed in the 1960s for use in the aircraft and automotive industries, and was first applied to dentistry a decade later.^25^ Dr Duret in the ’70 was the first to develop a dental CAD/CAM device aiming to make tooth restoration easier, faster, and more accurate. Since then, this technology has expanded considerably, in particular in orthodontics and cranio-facial surgery. One of the most described applications regards cranioplasty. A preliminary study published in 2015, Gordon Chad, with a retrospective review of 108 patients who underwent cranioplasty, has successfully demonstrated that immediate customized implant reconstructive techniques, are both safe and feasible for benign and malignant skull neoplasms.^26^, ^27^ Gordon himself has then published in 2022 a retrospective, 8-year study, on all consecutive patients undergoing single stage cranioplasty with customized implants compared with previous studies utilizing similar alloplastic implants, demonstrating that there are several advantages such as comprehensive resection and reconstruction plan using 3D models, shorter operative time, and better restoration of complex anatomy.^28^ This technology applies also to mandibular reconstruction with free fibula flap in patients with squamous cell carcinoma and mandibular osteoradionecrosis, achieving optimal stability and a good restoration of the mandibular shape without complications in 100 % of cases.^14^ The application described in the present article might be considered a step forward a more transversal fruition of a technology that in the near future may have a rapid expansion to other clinical application, including bioprinting.^29^ Indeed, in the present study the preliminary results of CAD-CAM technology applied to distal femur non-union treated with free fibula flap, custom made medial plating and maintenance of a stable lateral locking plate are reported. In the 2 described cases, the accurate multidisciplinary pre-operative planning and the senior surgeons’ multidisciplinary team were considered paramount to obtain the final satisfactory results achieved. Indeed, despite CAD-CAM technology proving its efficacy in reducing intraoperative difficulties, especially regarding combined vascularized grafting and medial plating, the procedure remains technically demanding. To our knowledge, the present work represents the first described case series in literature of the use of the CAD/CAM technology in planning distal femur non-union treatment. The technology offers an improved surgical precision defining the exact mechanical position and dimension of the custom-made medial additional plate and of the FFVG and its vascular pedicle. However, further studies are needed to confirm the validity and reproducibility of the described 105 treatment and to demonstrate the cost-savings offered by the application of CAD/CAM technology in the treatment of these particular non-unions. In our hands it allowed a safe, reliable and timesaving treatment, with a 100% resolution of the preoperative clinical condition and a return to everyday life with full weight bearing in both cases. The main limit of our study is the low number of patients involved, which is related to the rarity of the 110 condition treated.

## Ethics approval and consent to participate

All procedures performed in studies involving human participants were in accordance with the ethical standards of the institutional and/or national research committee and with the 1964 Helsinki declaration and its later amendments or comparable ethical standards. Written informed consent was obtained from the patients. The need for approval was waived by Università di Trieste Ethics committee/IRB.

## Consent for publication

Not applicable.

## Authors' contributions

All authors whose names appear on the submission.

1) made substantial contributions to the conception or design of the work; or the acquisition, analysis, or interpretation of data; or the creation of new software used in the work;
2) drafted the work or revised it critically for important intellectual content;
3) approved the version to be published; and
4) agree to be accountable for all aspects of the work in ensuring that questions related to the accuracy or integrity of any part of the work are appropriately investigated and resolved.

## Availability of data and material

All data generated or analysed during this study are included in this published article.

## Funding

No funding was received to assist with the preparation of this manuscript.

## Declaration of competing interest

The authors declare that they have no competing interests.

## List of abbreviation

CAD – CAM Technology: Computer-Aided Design-Computer-Aided Manufacturing
NSAID: Non-steroidal anti-inflammatory drugs
DICOM: Digital Imaging and Communications in Medicine
FVFG: Free vascularized fibula graft
MAC: Bone marrow aspirate concentrate
LLP: Locking long plate
